# The ALLAN trial: impact of early home-based palliative care on emergency care and hospitalisation in advanced gastrointestinal cancer patients

**DOI:** 10.1038/s41416-026-03444-8

**Published:** 2026-04-24

**Authors:** A. Bojesson, E. Brun, J. Eberhard, M. Segerlantz

**Affiliations:** 1 Department of Clinical Sciences, Oncology and Pathology, Faculty of Medicine, Lund University, Lund, Sweden; 2 Department of Oncology, Skåne University Hospital, Lund, Sweden; 3 Department of Clinical Sciences Lund, Respiratory Medicine, Allergology and Palliative Medicine, Institute for Palliative Care, Faculty of Medicine, Lund University, Lund, Sweden; 4 Department of Palliative Care and Advanced Home Health Care, Primary Health Care Skåne, Region Skåne, Lund, Sweden

**Keywords:** Gastrointestinal cancer, Palliative care

## Abstract

**Background:**

Patients with advanced gastrointestinal (GI) cancer experience a high symptom burden which frequently necessitates emergency care. Integration of early home-based specialised palliative care (SPC) with tumour-specific treatments may impact emergency healthcare use.

**Methods:**

At the initiation of palliative chemotherapy, patients with advanced GI cancer were randomised to early home-based SPC integrated with tumour-specific treatment, or tumour-specific treatment with SPC referral when needed. The aim was to compare quality of life in the two groups. Here we present secondary outcomes; number of emergency department visits, hospitalisations, days of inpatient care, the time from the last chemotherapy treatment to death, and the place of death between the study groups.

**Results:**

A total of 118 patients were randomised. Patients in the early SPC group had significantly fewer emergency department visits (median 1 versus 3), hospitalisations (median 1 versus 2), and inpatient care days (median 1.5 vs. 11.5) compared to the control group (*p* < 0.001). There was no significant difference between the study groups in either time between the last chemotherapy treatment and death, inpatient SPC or place of death.

**Conclusion:**

Early integration of home-based SPC in advanced GI cancer patients significantly reduces emergency healthcare use and hospitalisation.

**Clinical Trial Registration:**

ClinicalTrials.gov (ref: NCT02246725).

## Introduction

Palliative care for cancer patients is commonly seen as end of life (EoL) care, and patients are usually referred late in the disease trajectory [1], leading to missed opportunities for comprehensive palliative care. Specialised palliative care (SPC) may be needed for complex patients; this involves a multidisciplinary team including specialists in palliative medicine. The aim of SPC for patients with advanced cancer is to alleviate symptoms and distress associated with the burden of the disease.

Studies have shown that the addition of SPC to tumour-specific treatment can decrease disease- and treatment-related symptoms, reduce anxiety and depression, and result in an increased quality of life (QoL) compared to patients receiving tumour-specific treatment alone [2–7]. We have previously reported the positive impact on QoL in the randomised ALLAN trial comparing palliative chemotherapy +/- early integration of home-based SPC for patients with advanced gastrointestinal (GI) cancer [8]. Nevertheless, there is a scarcity of randomised clinical trials investigating the impact of early integration of home-based SPC on issues such as the need for emergency department visits and hospitalisation, chemotherapy use close to death, and place of death.

### Palliative care and emergency care needs

Intense medical interventions for cancer patients in the last weeks of life, such as intravenous chemotherapy, emergency department visits, hospital admissions, including intensive unit care, are indicators of poor QoL near the EoL [9, 10]. These hospitalisations, including emergency department visits, often include diagnostic procedures such as imaging and blood sampling, which are inappropriate at the EoL and contribute to a poorer QoL for patients near death [11].

A retrospective cohort study by Hui et al. examined how the timing of a palliative care referral influenced the quality of end-of-life care in 366 patients with advanced cancer who received a palliative care consultation prior to death. The study categorised patients into two groups: those referred to palliative care more than 3 months before death and those referred closer to death. Patients who were enrolled in palliative care for a longer duration had fewer emergency department visits (39% vs. 68%) and hospitalisations in the last 30 days of life (48% vs. 81%); they were also less likely to die in a hospital (17% vs. 31%) compared to those referred to palliative care 3 months or less before death [12]. Conversely, a Cochrane review from 2021 found no conclusive evidence that home-based palliative care reduced unplanned hospital admissions for patients at the EoL [13].

### Palliative care and chemotherapy use close to death

Chemotherapy within the final 30 days before death is seen as futile, and it carries the risk of decreasing patients’ QoL at the EoL [14, 15]. Greer et al. observed that patients with metastatic non-small cell lung cancer who were randomly assigned to early integration of SPC had a risk-reduction of 50% for receiving intravenous chemotherapy during the last 2 months of life compared to patients randomised to standard oncological care alone [16]. In a retrospective study involving 681 cancer patients, researchers examined factors influencing the administration of chemotherapy in the final 30 days of life. They found that referral to palliative care was linked to a decreased risk of receiving chemotherapy in the last month of life (odds ratio: 0.35, *p* < 0.001). Notably, the risk was even lower for patients who received an early referral to palliative care ( >90 days before death) [17]. A retrospective study by Ekström et al. showed that patients enrolled in SPC for more than 30 days before death had a longer period of time between the last course of palliative chemotherapy and death (median 73 days versus 44 days, *p* <  0.001) without any difference in overall survival (OS) [18].

### Palliative care and place of death

Patients often express a preference to spend their final days at home, and to die there, rather than in a hospital setting [19]. A Danish randomised controlled trial, including 205 patients with late-stage heart- or lung disease or with advanced cancer, investigated whether the actual place of death differed between patients who discussed their preferred place of death with a physician in an advanced care planning meeting and those who did not [20]. Among patients who participated in such a meeting, 40% died at home, compared to 17% of patients not involved in such meetings (*p* = 0.013). We have earlier shown in patients diagnosed with advanced pancreatic cancer that only 2% of patients who were ever enrolled in SPC died at a hospital, whereas for patients who were never enrolled, the corresponding figure was 84% [18].

#### Aim

In the primary report of a randomized controlled trial (ALLAN) on early integration of SPC for patients with GI cancers undergoing palliative chemotherapy we have reported on the positive effect on QoL [8]. The aim of the present study is to assess the impact of early integrated home-based SPC on the number of emergency department visits, number of hospitalisations, length of inpatient care, time between the last chemotherapy treatment and death, and place of death for the patients, all secondary aims of the mentioned RCT.

## Material and methods

This prospective randomised clinical trial (ALLAN) was conducted at a tertiary cancer centre in the southern Swedish healthcare region, and enrolled patients from December 18, 2014, to April 29, 2021. The study included patients in the catchment area of two major SPC units, serving approximately 0.5 million residents. The last date of follow-up was March 1, 2023. In this article we report the secondary outcomes of the study, following the previously published findings [8].

### Study protocol, inclusion criteria, and exclusion criteria

Ambulatory adult patients ( >18 years) with a WHO performance status of 0–2 and with upper GI cancer (oesophageal, gastric, hepatobiliary, and pancreatic cancer) eligible for first-line palliative chemotherapy treatment and patients with lower GI cancer (colorectal cancer) eligible for second-line palliative chemotherapy treatment were recruited to this nonblinded randomised controlled trial. The patients with colorectal cancer commencing second-line treatment were expected to have a similar survival time to those with upper GI cancers.

On the first consultation at the Oncology Department patients were randomised 1:1 to oncological treatment +/- early integration of home-based SPC. Patients in the control arm (standard of care) were referred to SPC when in need. In the active arm, the first SPC consultation occurred within 6 weeks after randomisation and further consultations took place at least monthly afterward. Patients who already were on palliative chemotherapy except for patients with colorectal cancer, or patients with neuroendocrine tumors, or patients already enrolled in SPC were excluded and not offered participation in the study.

### Demographics and tumour-specific data

Demographic data were recorded, including age, sex, diagnosis, date of randomisation, performance status at randomisation, chemotherapy regimens, date of last chemotherapy treatment, and date of death.

### Intervention

A structured visit report was used to evaluate individuals in the active study group during a home visit, following the study protocol, at 6-week intervals (Appendix 1). Patients were asked about their understanding of their disease, treatment, prognosis, daily activities, and QoL. In addition, at the same visit, a comprehensive systematic symptom assessment was performed using the Integrated Palliative Care Outcome Scale (IPOS) [21].

Patients in the active study group received tumour-specific treatment at the outpatient unit in the Department of Oncology and were presented to the multi-professional SPC team, including palliative care physicians, palliative care nurses, dieticians, occupational therapists, counsellors, and physiotherapists, within 6 weeks of randomisation. Patients were offered home consultations with 24/7 availability and admittance for inpatient care if needed. The SPC team delivered advanced home care with the possibility of administering intravenous fluids, including antibiotics, blood products, and nutritional support. The team also conducted assessments of symptoms, monitored pain management, and observed patients for potential side effects related to medications. The home-based SPC team was able to collect blood samples and refer patients for radiological examinations to support clinical decision-making. In addition, side effects related to oncological treatments were assessed in close collaboration with the treating oncologist. Information on the number of contacts, homecare visits, and telephone calls with the SPC team was extracted from patients’ original medical records. Patients in the control group were subjected to a SPC referral when deemed needed.

### Emergency ward visits and the need for inpatient care

Data on emergency department visits, hospitalisations, duration of inpatient care, and days spent as inpatients in SPC were retrieved from patients’ medical records. Information on emergency department visits and inpatient care was collected from the time of the trial inclusion.

### Statistics

Numerical variables are presented in this article as median and range, and categorical variables are presented as absolute and relative frequencies. Emergency healthcare utilisation was compared between the two arms using the Mann-Whitney U test and visualised with boxplots. The median difference was estimated with the Hodges–Lehmann procedure and presented with a 95% confidence interval. Median OS both from inclusion and from last chemotherapy treatment was estimated using the Kaplan-Meier method, with patients alive at the last follow-up (March 1, 2023) being censored at that date. Survival in the two arms was compared with the log-rank test. The proportion of patients receiving palliative chemotherapy within 30 days before death was compared using the chi-squared test. All statistical analyses were performed with R 4.2.2.

## Results

### Demographics

**Fig. 1:**
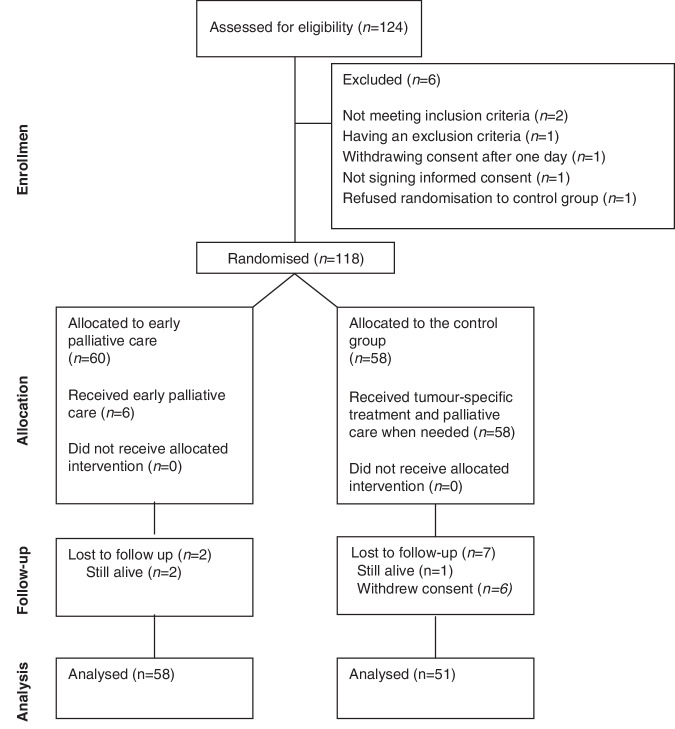
Consort diagram.

Of the 124 patients who were initially included, 6 were excluded due to not meeting the inclusion criteria (*n* = 2), meeting an exclusion criterion (*n* = 1), withdrawing consent (one day after randomisation) (*n* = 1), refusing randomisation to the control group (*n* = 1), and not signing informed consent (*n* = 1)(see consort diagram in Fig. 1). The remaining 118 patients comprised 55 women with a median age of 70 (45–83) years and 63 men with a median age of 74 (49–85) years. Six patients subsequently withdrew their consent and were included in the analysis up to the point of withdrawal, except for the ongoing survival analysis. The participating patients had been diagnosed with pancreatic cancer (*n* = 66), hepatobiliary cancer (*n* = 25), gastric cancer (*n* = 11), colon cancer (*n* = 9), or oesophageal cancer (*n* = 7). Baseline performance status (PS) was distributed among the active study group and the control group as follows: 30% versus 45% for PS scores of 0, 47% versus 36% for PS scores of 1, and 23% versus 19% for PS scores of 2, respectively. Patients in the active group and the control group received a median of 6.0 (0–34) and 6.0 (0–33) cycles of palliative chemotherapy, respectively (see Table 1 for demographic data).

**Table 1:** Baseline characteristics of the study participants, specialised palliative care use data, and overall survival.

Variable	Whole study cohort (*n*=118)	Active group (*n*=60)	Control group (*n*=58)
Age, median [range]	71.5 [45, 85]	71 [51, 83]	72 [45, 85]
Female sex, *n* (%)	55 (46.6)	24 (40.0)	31 (53.4)
Cancer diagnosis, *n* (%)			
Pancreatic	66 (55.9)	35 (58.3)	31 (53.4)
Hepatobiliary	25 (21.2)	11 (18.3)	14 (24.1)
Gastric	11 (9.3)	7 (11.7)	4 (6.9)
Colorectal	9 (7.6)	4 (6.7)	5 (8.6)
Oesophageal	7 (5.9)	3 (5.0)	4 (6.9)
WHO performance status, *n* (%) ^a^			
0	44 (37.3)	18 (30.0)	26 (44.8)
1	49 (41.5)	28 (46.7)	21 (36.2)
2	25 (21.2)	14 (23.3)	11 (19.0)
Palliative chemotherapy lines, *n* (%) ^b^	**(*n*=112)**	**(*n*=60)**	**(*n*=52)**
0	2 (1.8)	1 (1.7)	1 (1.9)
1	66 (58.9)	34 (56.7)	32 (61.5)
2	27 (24.1)	14 (23.3)	13 (25.0)
3	17 (15.2)	11 (18.3)	6 (11.5)
Anticancer therapy, *n* (%) ^c^	**(*n*=110)**	**(*n*=59)**	**(*n*=51)**
Nab-paclitaxel/gemcitabine	25 (22.7)	14 (23.7)	11 (21.6)
FOLFIRINOX	24 (21.8)	12 (20.3)	12 (23.5)
Gemcitabine	24 (21.8)	13 (22.0)	11 (21.6)
Combination chemotherapy	14 (12.7)	8 (13.6)	6 (11.8)
FOLFOX	12 (10.9)	8 (13.6)	4 (7.8)
GEMOX	8 (7.3)	2 (3.4)	6 (11.8)
Single-agent chemotherapy	3 (2.7)	2(3.4)	1 (2.0)
Number of chemotherapy cycles, median [range]	6.0 [0.0, 34.0]	6.0 [0.0, 34.0]	6.0 [0.0, 33.0]
	**(*n*=118)**	**(*n*=60)**	**(*n*=58)**
Days enrolled in SPC, median [range]		167 [4, 987]	39 [0, 1152]
Assessments by SPC, median [range]			
Physician		10 [1, 34]	2 [0, 24]
Nurse		36 [0, 330]	13 [0, 210]
Telephone		22 [0, 79]	4 [0, 135]
Survival in months, median (95% CI)	7.6 (6.0–10.2)	6.6 (4.9–10.7)	8.7 (6.5–12.2)

*FOLFIRINOX* oxaliplatin, irinotecan, leucovorin, and fluorouracil, *FOLFOX* 5-fluorouracil, oxaliplatin, and leucovorin, *GEMO*X gemcitabine and oxaliplatin, Nab-paclitaxel, albumin-bound paclitaxel.

^a^WHO performance status of 0 indicates that the patient is asymptomatic, 1 indicates that the patient is symptomatic but fully ambulatory, and 2 indicates that the patient is symptomatic and in bed less than 50% of the day.

^b^Six patients withdrew consent and were excluded from this analysis.

^c^Two patients did not start palliative chemotherapy; six withdrew consent and were excluded from this analysis.

### Length of enrolment and intensity of contact with the SPC

Patients in the active group were admitted to the SPC unit for a median of 167 (4–987) days. In the control group, all but eight patients were admitted to SPC at some time point during the disease trajectory, with a median of 39 (0–1152) days of enrolment.

The active and control groups had a median of 10 (1–34) versus 2 (0–24) homecare visits from an SPC team physician, a median of 36 (0–330) versus 13 (0–210) visits from an SPC nurse or other healthcare professional, and a median of 22 (0–79) versus 4 (0–135) telephone calls with any healthcare professional, respectively.

### Emergency department visits and the need for inpatient hospital care

**Fig. 2:**
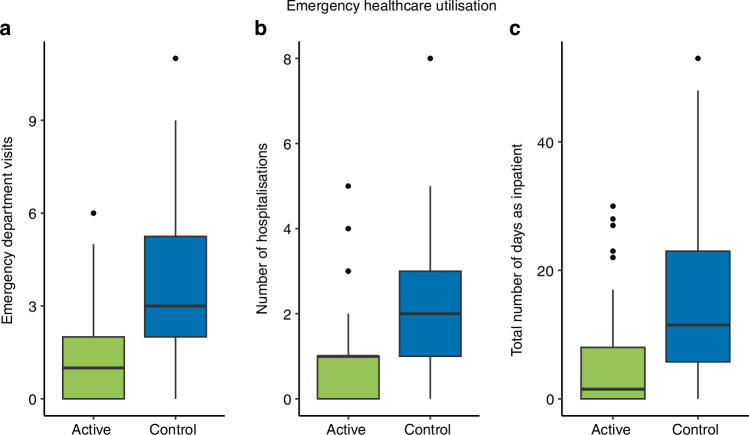
Box plots showing emergency department visits and inpatient hospital care. **a** Numbers of emergency department visits in the two study groups. **b** Numbers of hospitalisation episodes in the two study groups. **c** Numbers of days of inpatient hospital care in the two study groups. The interquartile range represents 25–75% of the patients at each measuring point, the whiskers represent 1.5 times the interquartile range, and the solid black circles indicate outliers.

Patients in the active study group had a median of 1 (0–6) visit to the emergency department, a median of 1 (0–5) hospitalisation episode, and a median of 1.5 (0–30) days of inpatient hospital care. Patients in the control group utilised significantly more emergency healthcare and inpatient hospital care, with a median of 3 (0–11) visits to the emergency department, a median of 2 (0–8) hospitalisation episodes, and a median of 11.5 (0–33) days of inpatient hospital care (*p* = <0.001) (Fig. 2 and Table 2).

**Table 2:** Emergency care utilisation and admissions to hospital and the inpatient palliative care ward.

Healthcare utilisation Median [min-max]	Active group (*n* = 60)	Control group (*n* = 58)	Difference in median (95% CI)	*p*-value
Emergency department visits	1 [0−6]	3 [0−11]	−2 (−3; −1)	<0.001
Number of admissions to hospital	1 [0−5]	2 [0−8]	−1 (−1; −1)	<0.001
Number of inpatient hospital days	1.5 [0−30]	11.5 [0−53]	−8 (−11; −4)	<0.001
Number of admissions to inpatient SPC	1 [0−3]	1 [0−7]	0 (−0; 0)	0.274
Number of days of inpatient SPC	5.5 [0−200]	4.5 [0−155]	0 (0; 3)	0.538
Number of admissions to hospital and inpatient SPC	2 [0−6]	3 [0−15]	−1 (−1; 0)	0.003
Number of days as an inpatient in a hospital and SPC	10 [0−203]	17.5 [0−185]	−7 (−12; −2)	0.013

### Need for inpatient SPC

There was no difference in use of inpatient SPC between the study groups. Patients in the active study group had a median of 1 (0–3) admission to inpatient SPC and a median of 5.5 (0–200) days as inpatients. Patients in the control group had a median of 1 (0–7) admission to inpatient SPC and a median of 4.5 (0–155) days as inpatients (*p* = 0.274 and *p* = 0.538, respectively) (Table 2).

### Total need for inpatient SPC and hospital care

Patients in the active group had a median of 10 (0–203) days of inpatient care compared to 17.5 (0–185) days in the control group (*p* = 0.013). Patients in active group had a median of 2 (0–6) admissions to hospital and the inpatient SPC compared to 3 (0–15) in the control group (*p* = 0.003) (Table 2).

### Chemotherapy treatment close to death

**Fig. 3:**
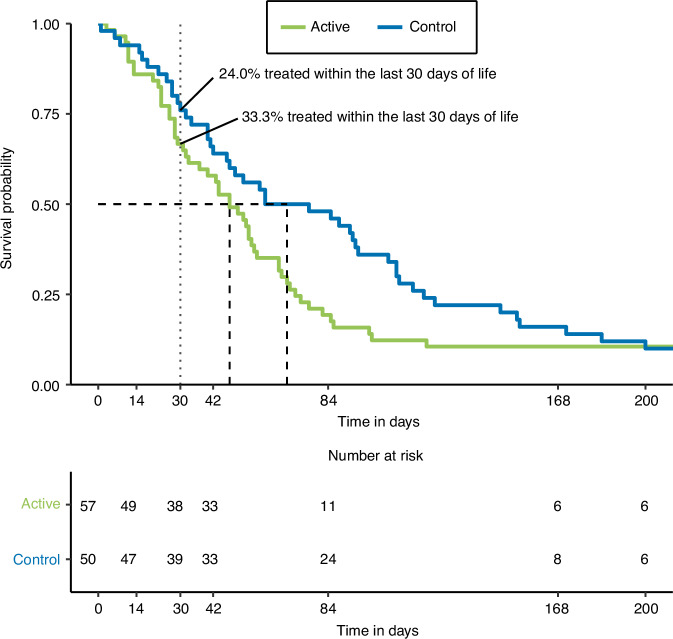
Kaplan-Meier curve showing the time between last palliative chemotherapy treatment and death. There was no statistically significant difference between the two groups in the time between the last chemotherapy received and death (*p* = 0.053).

There was no statistically significant difference between the groups in the time from the last chemotherapy treatment to death. Patients in the active study group lived a median of 48 days (95% CI: 33–58) from the last chemotherapy treatment versus 69 days (95% CI: 47–109) for patients in the control group (*p* = 0.053). In the active group, 33% of patients received palliative chemotherapy in the last 30 days of life versus 24% in the control group (*p* = 0.28) (Fig. 3).

### Overall survival

At the time of analysis, 3 patients were still alive. No statistically significant differences in OS were found between the study groups. The median OS for the cohort of 118 patients was 7.6 months (95% CI: 6.0–10.2). When comparing the groups, median OS was 6.6 months (95% CI: 4.9–10.7) in the active study group versus 8.7 months (95% CI: 6.5–12.2) in the control group (*p* = 0.675).

### Place of death

Upon conclusion of the study, 58 out of 60 patients in the active group had died. Among these, 40% (*n* = 23) died at home, while 57% (*n* = 33) died in the inpatient SPC ward and 3% (*n* = 2) died in the hospital. Similarly, at the termination of the study, 51 patients in the control group had died, while six individuals had withdrawn their consent. Of the deceased, 41% (*n* = 21) died at home, 45% (*n* = 23) died in the inpatient SPC ward, 12% (*n* = 6) died in the hospital, and 2% (*n* = 1) died in a facility for assisted living. Among the 8 patients in the control group who were never referred to SPC, 75% (*n* = 6) died in the hospital and 25% (*n* = 2) at home.

## Discussion

We found, in GI-cancer patients randomized to early integration of home-based SPC, fewer emergency department visits and fewer days of inpatient care, compared to patients in the control group, receiving SPC later, or not at all, in the disease trajectory. The difference in emergency healthcare utilisation was of clinical importance, with patients in the active group having fewer (two days less) visits to the emergency department than patients in the control group, as well as ten fewer days as inpatients. Even when taking the inpatient SPC into account the control group spent in total seven more days as inpatients compared to patients in the active group. Moreover, fewer contacts with emergency healthcare reduces the risk of futile medical investigations [11].

In contrast to our results, a Cochrane review of home-based palliative care found no statistically significant reduction in emergency department visits for patients receiving home-based palliative care at the EoL compared to patients without these services [13]. In a heterogeneous patient cohort, Brumley et al. included 298 patients with either advanced cancer, chronic heart failure, or chronic obstructive pulmonary disease, with an estimated life expectancy of 12 months, and randomised them to home-based palliative care or standard of care including general palliative care. Patients randomised to home-based palliative care spent 4.4 days less in the hospital, and only 20% visited the emergency department, in contrast to 33% of patients in the standard-of-care group [22].

Given the diversity between the extent and model of palliative care services, it is hard to compare outcomes in published studies. To our knowledge, only a few studies have investigated home-based SPC as an intervention and its effects on emergency care utilisation. For example, the abovementioned Cochrane review from 2021 found only 3 studies in their analysis of how home-based palliative care affects emergency care use [13]. In the present study, contact with SPC was intensive and resource-demanding. The reduced need for emergency healthcare is partly self-explanatory, as the active group of patients had around-the-clock availability of the SPC team, with a median of 2 home visits per week in addition to telephone consultations. However, our findings align with the hypothesis that early integration of a home-based SPC team, tailored to the needs of patients with advanced GI cancer, can effectively mitigate emergency healthcare visits while providing essential support for those choosing EoL care at home. We did not analyse for differences in emergency department visits in the last month of life as nearly all study patients (86%) by then were admitted to the SPC team. In addition, we did not stratify utilisation by proximity to death, in order to capture the overall burden of care throughout the disease trajectory and to avoid survival bias.

Palliative chemotherapy can result in symptom control and offer maintained autonomy for patients in the palliative situation [23]. However, the American Society of Clinical Oncology states that chemotherapy close to death, with a lack of evidence of clinical value, is a widespread and an unnecessary practice in oncology [24]. Also, chemotherapy close to death entails a false hope of prolonged life and detracts from a focus on prioritisation and care planning at the EoL. Nevertheless, our trial showed no statistically significant differences between the study groups in either chemotherapy treatment intensity or the interval between the last palliative chemotherapy treatment and death. This result is surprising, as previous studies found that patients admitted early to SPC had a longer time between the last palliative chemotherapy treatment and death [16, 18]. For example, Greer et al. showed that patients randomised to early integration of SPC had 64 days between the last palliative chemotherapy treatment and death, versus 40 days in the standard of care group (*p* = 0.02) [16]. Our active group received palliative chemotherapy a median of 48 days from death compared to 69 days in the control group (*p* = 0.053), with no statistically significant difference in OS. The numerical difference in days and an almost statistically significant p-value regarding the time between the last chemotherapy treatment and death suggests that patients in the active arm received palliative chemotherapy closer to death. A possible explanation could be that the comprehensive symptom management and supportive care provided by the SPC team contributed to a good functional status among patients in the active group. Consequently, these patients were offered palliative chemotherapy for a longer period of time compared to those in the control group, which is a possible explanation for the finding of the shorter time between the last course of chemotherapy treatment and death. This reasoning is supported by our previously published results with the improved QoL in the active group 24 weeks after randomisation compared to patients in the control group [8].

The median time of enrolment in SPC for the active and control group was 167 days and 39 days, respectively, and only 8 patients (14%) in the control group were never enrolled in SPC. A systemic overview performed in 2017 showed that an average of 60% of terminally ill cancer patients prefer to die at home [25]. We examined the place of death, and not the preferred place of death, and observed a similar proportion of patients dying at home in both study groups, with 40% in the active group and 41% in the control group. This was expected as many as 86% of patients in the control group eventually were enrolled in SPC. Our results are in line with the abovementioned Danish randomised controlled trial, where 40% of patients who had been involved in advanced care planning died at home [20].

During the final days before death, the wish to die at home may turn out to be unrealistic. In the present study, 52% of all SPC-included patients died at the SPC ward, implying that emergency department visits had been avoided in favour of admittance to in-house SPC or continued homecare. This assertion finds further validation in the fact that 6 of the 8 patients who were never referred to SPC died in the hospital.

Our randomised trial is subject to several limitations. We do not have an exclusion log, which would have been of interest, as the inclusion period was long relative to the expected flow of new patients at the Department of Oncology. There may have been some selection bias, as we saw an unexpectedly low proportion of individuals with colorectal cancers receiving second-line palliative therapy. It is possible that the recruiting physicians found it more difficult to mark a boundary between curative-intent and palliative-intent treatment in patients with colorectal cancer, compared to patients with advanced pancreatic cancer. One of the challenges of this study was to inform patients, at their first appointment with the oncologist, about the palliative intent of the treatment while at the same time providing hope with a tumour-directed therapy. We believe this may have hampered our colleagues’ involvement in inviting patients to the study. Furthermore, the power of this trial was calculated on our primary outcome and not the pre-specified secondary outcomes presented in this article.

Our study possesses several strengths, as our SPC model allowed us to study the impact of high-quality home-based care in a group of patients with a high burden of symptoms and a limited expected survival time. Aggressive EoL care, such as emergency department visits, hospital care, and late-stage chemotherapy, is detrimental to the QoL of patients and families, and can raise false hopes for recovery. It also represents futile overuse of healthcare expenditures. Our model of home-based and multiprofessional SPC reduced emergency healthcare use even though nearly 90% of the patients in the control group were eventually admitted to SPC before death.

## Conclusion

This prospective randomised study provides evidence supporting the benefit of early integration of home-based SPC alongside tumour-specific treatment for patients diagnosed with advanced GI cancers in less utilisation of emergency care resources and hospitalisations.

## Supplementary information

Consort diagram, Figure 1

Appendix 1

## Data Availability

The datasets generated and analysed during the current study are not publicly available due to the data protection law in Sweden. The datasets are available from the corresponding author upon reasonable request.
