# Supplementary material for: The ALLAN trial: impact of early home-based palliative care on emergency care and hospitalisation in advanced gastrointestinal cancer patients
Source: Br J Cancer. 2026 Apr 24;135(2):248–54. doi: 10.1038/s41416-026-03444-8 (PMC13310842; doi:10.1038/s41416-026-03444-8)
Supplement: Supplementary file 1 — Consort diagram, Figure 1 [file 41416_2026_3444_MOESM1_ESM.doc]

Consort diagram.

Assessed for eligibility (*n*=124)

Excluded (*n*=6)

Not meeting inclusion criteria (*n*=2)

Having an exclusion criteria (*n*=1)

Withdrawing consent after one day (*n*=1)

Not signing informed consent (*n*=1)

Refused randomisation to control group (*n*=1)

**Enrollment**

Randomised (*n*=118)

#

Allocated to early palliative care

(*n*=60)

Received early palliative care (*n*=60)

Did not receive allocated intervention (*n*=0)

Allocated to the control group

(*n*=58)

Received tumour-specific treatment and palliative care when needed (*n*=58)

Did not receive allocated intervention (*n*=0)

**Allocation**

**Follow-up**

Lost to follow up (*n*=2)

Still alive (*n*=2)

Lost to follow-up (*n*=7)

Still alive (n=1)

Withdrew consent (*n=6)*

**Analysis**

Analysed (n=58)

Analysed (n=51)
